# A stress-responsive bZIP transcription factor *OsbZIP62* improves drought and oxidative tolerance in rice

**DOI:** 10.1186/s12870-019-1872-1

**Published:** 2019-06-17

**Authors:** Shiqin Yang, Kai Xu, Shoujun Chen, Tianfei Li, Hui Xia, Liang Chen, Hongyan Liu, Lijun Luo

**Affiliations:** 1 College of Plant Science & Technology, Huazhong Agricultural University, Wuhan, 430070 China; 2 Shanghai Agrobiological Gene Center, Shanghai, 201106 China

**Keywords:** Abscisic acid, Drought tolerance, *OsbZIP62*, Oxidative tolerance, Rice, Transcription factor

## Abstract

**Background:**

Drought is a major abiotic stress factor that influences the yield of crops. Basic leucine zipper motif (bZIP) transcription factors play an important regulatory role in plant drought stress responses. However, the functions of a number of bZIP transcription factors in rice are still unknown.

**Results:**

In this study, a novel drought stress-related bZIP transcription factor, *OsbZIP62*, was identified in rice. This gene was selected from a transcriptome analysis of several typical rice varieties with different drought tolerances. *OsbZIP62* expression was induced by drought, hydrogen peroxide, and abscisic acid (ABA) treatment. Overexpression of *OsbZIP62-VP64* (*OsbZIP62V*) enhanced the drought tolerance and oxidative stress tolerance of transgenic rice, while *osbzip62* mutants exhibited the opposite phenotype. OsbZIP62-GFP was localized to the nucleus, and the N-terminal sequence (amino acids 1–68) was necessary for the transcriptional activation activity of OsbZIP62. RNA-seq analysis showed that the expression of many stress-related genes (e.g., *OsGL1*, *OsNAC10*, and *DSM2*) was upregulated in *OsbZIP62V* plants. Moreover, OsbZIP62 could bind to the promoters of several putative target genes and could interact with stress/ABA-activated protein kinases (SAPKs).

**Conclusions:**

*OsbZIP62* is involved in ABA signalling pathways and positively regulates rice drought tolerance by regulating the expression of genes associated with stress, and this gene could be used for the genetic modification of crops with improved drought tolerance.

**Electronic supplementary material:**

The online version of this article (10.1186/s12870-019-1872-1) contains supplementary material, which is available to authorized users.

## Background

Rice is one of the most important grain crops and the most water-consuming crop (approximately 65% of agricultural water) [1, 2]. Due to environmental pollution and climate change, plants frequently encounter various abiotic stresses (such as drought, heat, and salt) that influence their growth and development during the growth process. Drought is one of the major stress factors that severely affects plant growth and results in a series of physiological and biochemical changes [3]. In response to drought stress, plants have evolved a set of very complex and effective strategies that include regulating stomatal closure to reduce plant transpiration and water loss; accumulating osmolytes (such as inorganic ions, organic solutes and sugars) to maintain the osmotic balance of cells; and increasing superoxide dismutase (SOD), catalase (CAT) and other antioxidants to maintain cell membrane stability [4]. These plant physiological and biochemical responses are achieved mainly via stress signal transduction and the transcriptional regulation of downstream stress-related genes [1].

ABA is one of the most widely studied plant hormones that modulate both plant growth and stress responses [5–7]. In recent years, studies have shown that ABA helps plants cope with abiotic stress mainly due to the participation of their receptors. The main concept is as follows: ABA combines with PYR/PYL/RCARs proteins to form a compound, which then interacts with protein phosphatase 2Cs (PP2Cs) to inhibit the activity of PP2Cs, thereby relieving the inhibition of PP2C on SnRK2s. SnRK2s subsequently activate downstream transcription factors by phosphorylation [8–10]. Among these transcription factors, bZIPs compose one of the transcription factor families that has been extensively studied. It has been found that bZIP transcription factors play various roles in plant growth and stress responses, such as seed maturation, disease resistance, and drought resistance [1, 11, 12]. There are approximately 110 bZIP transcription factors in *Arabidopsis thaliana*, some of which have been identified [13–15]. On the basis of their structure and function, plant bZlP transcription factors are classified into 10 subfamilies [14]. The bZIP transcription factors of different groups play various roles in the abiotic stress response. For example, many members of the group C bZIP transcription factors, known as the *ABF/AREB/ABI5* subfamily, participate in ABA signal transduction and the stress response [16, 17]. *ABF1* is mainly involved in ABA and low-temperature stress responses, and *ABF2/AREB1* and *ABF4/AREB2* are mainly involved in ABA, drought, high-salt, heat and oxidative stress responses [18–20].

There are 89 bZIP transcription factors in rice [21, 22]. Studies have shown that several members of the third subfamily, such as *OsbZIP23*, *OsbZIP46*, and *OsbZIP72*, function in rice stress responses [23–25]. Overexpression of *OsbZIP23* in transgenic rice plants significantly improved rice drought resistance and salt tolerance and increased sensitivity to ABA [23, 26]. *OsbZIP46*, which encodes a transcription factor and is homologous to *OsbZIP23*, plays a very important role in mediating ABA signalling and drought resistance. Overexpression of a constitutively active form of *OsbZIP46* significantly increased the drought resistance of rice [25, 27]. *OsbZIP72* was identified by yeast one-hybrid screening. Its transcriptional activity mainly depends on the amino acid sequence of its N-terminus. Overexpression of *OsbZIP72* in rice plants can also enhance the ABA sensitivity and drought resistance [24, 28].

Though the functions of several bZIP genes in rice have already been determined in terms of stress resistance, a number of bZIP genes have not been identified in rice. In this study, we identified a novel bZIP transcription factors, *OsbZIP62*, and the results showed that *OsbZIP62* is a key regulator in rice drought and oxidative stress responses and has potential application in genetically modified crops with improved drought tolerance.

## Results

### *OsbZIP62* encodes a bZIP protein that belongs to the third subfamily of bZIP transcription factors in rice

In our previous study, the transcriptomes of several typical rice varieties with different drought tolerances were analyzed (unpublished). Some drought tolerance candidate genes whose expression was significantly correlated with stress physiological indices (e.g., osmotic potential) were selected (data not shown). Among these genes, *OsbZIP62* was selected for further study. *OsbZIP62* encodes a putative bZIP transcription factor [22]. Protein sequence analysis demonstrated that OsbZIP62 contains a typical basic region and leucine zipper domains (Additional file 1: Figure S1). Phylogenetic analysis of the third subgroup members of Arabidopsis and rice bZIP transcription factors showed that OsbZIP62, OsbZIP72 (LOC_Os09g28310), OsbZIP46 (LOC_Os06g10880) and OsbZIP23 (LOC_Os02g52780) were distributed on the same branch (Additional file 1: Figure S2) and that OsbZIP62 was near OsbZIP72, whose function is related to ABA and drought [24]. These results suggested that *OsbZIP62* might play a similar role in plant ABA and drought responses.

### Expression pattern of *OsbZIP62* under different stresses

**Fig. 1:**
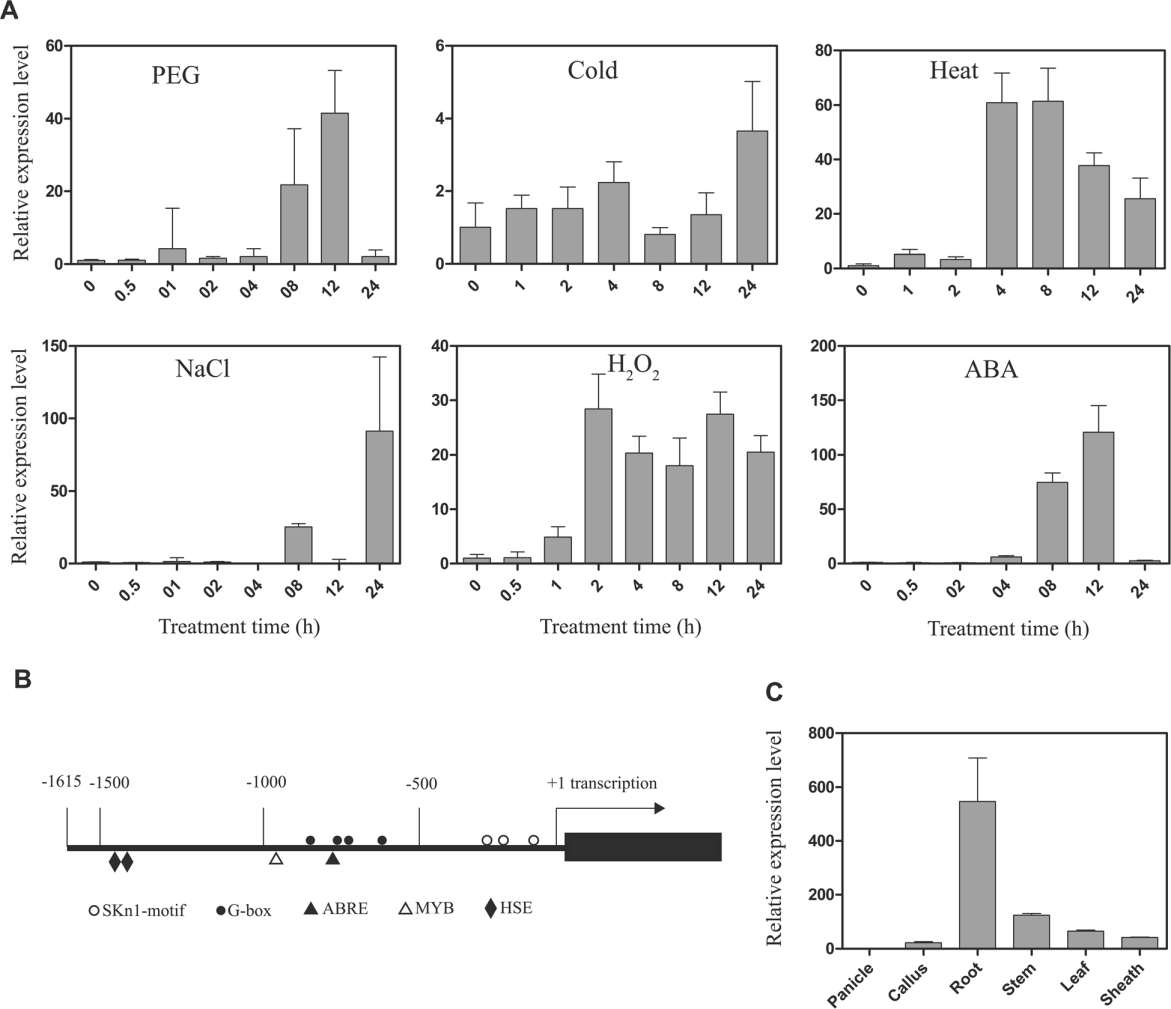
Expression pattern analysis of OsbZIP62 under abiotic stresses and hormones. **a**, Relative expression level of *OsbZIP62* under various stresses, including PEG6000 (15%), cold (4 °C), heat (42 °C), NaCl (150 mM), H_2_O_2_ (1%) and ABA (100 μM). **b**, Main stress-related cis-acting elements in the *OsbZIP62* promoter region. **c**, Relative expression level of *OsbZIP62* in different tissues (roots, stems, leaves, sheaths, panicles and calli) of rice plants under normal condition. The data represent the means ± SE (*n* = 3). The transcription factor site recognition information resource is available (http://bioinformatics.psb.ugent.be/ebtools/plantcare/html/)

To investigate and predict the function of *OsbZIP62*, the expression pattern of *OsbZIP62* under different abiotic stresses and phytohormone treatments was investigated by qPCR. *OsbZIP62* expression was significantly induced by polyethylene glycol (PEG)-simulated drought stress, oxidative stress (induced by H_2_O_2_), heat stress, salt stress, and ABA treatment and was slightly induced by cold stress (Fig. 1a). Bioinformatic analysis showed that there were many predicted stress response-related cis-elements, such as G-box recognition site (4 hits), MYB recognition site (1 hit), ABRE element (1 hit) and HSE recognition site (2 hits), in the promoter of *OsbZIP62* (Fig. 1b).

In addition, we also measured the tissue specificity of this gene in different tissues of rice by qPCR. The results showed that *OsbZIP62* was expressed in various tissues, but the expression level was high in the roots and relatively low in the panicles (Fig. 1c).

### Transactivation activity and subcellular localization of OsbZIP62

**Fig. 2:**
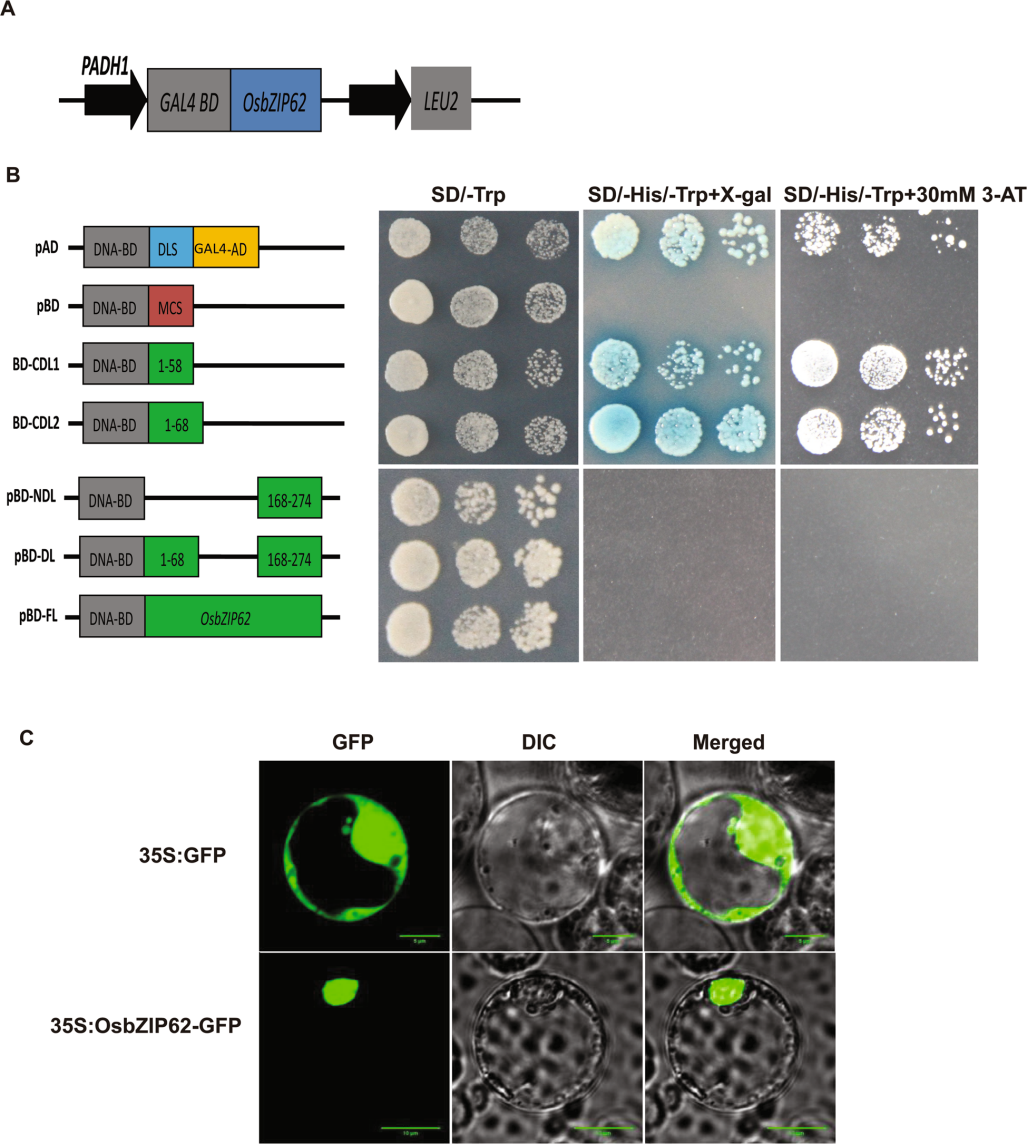
Transactivation assay and subcellular localization of OsbZIP62 protein. **a**, Transactivation activities of the different portions of OsbZIP62 were checked in yeast. BD: GAL4 DNA-binding domain; FL: full length; CDL: C-terminus deletion domain; and NDL: N-terminus deletion domain. **b**, Subcellular localization of OsbZIP62 in rice protoplasts. GFP and OsbZIP62-GFP under the control of the CaMV35S promoter separately transiently expressed in rice protoplasts

The transcriptional activation activity of the OsbZIP62 protein was determined in yeast. The full length of OsbZIP62, which was fused to the DNA-binding domain of GAL4 (pBD-FL), displayed no transactivation activity in yeast (Fig. 2a and b). Therefore, a series of OsbZIP62 deletions of part of the fragment were tested. The results showed that the expression of the reporter gene could be activated when the C-terminus (amino acids 168–274) was absent (pBD-CDL1 and pBD-CDL2), indicating that the amino acid sequences at the N-terminus (amino acids 1–68) are required for the transactivation activity of OsbZIP62 (Fig. 2b).

An OsbZIP62 protein and green fluorescent protein (GFP) under the control of the CAMV35S promoter were constructed and introduced into rice protoplasts. As shown in Fig. 2c, fluorescence of OsbZIP62-GFP was observed in the nucleus, indicating that OsbZIP62 is a nuclear-targeted protein.

### Increased ABA sensitivity of *OsbZIP62V* transgenic plants

Because the full length of OsbZIP62 had no transactivation activity, it is possible that overexpression of *OsbZIP62* is unable to lead to the presumed phenotype. Therefore, we selected *OsbZIP62V* plants for phenotypic investigation. In these plants, *OsbZIP62* coding sequences are fused to a VP64 (tetrameric repeats of VP16) universal transcriptional activation module to maintain transcriptional activation (Additional file 1: Figure S3A). The expression level of *OsbZIP62V* was significantly higher in transgenic plants than in wild-type (WT) plants, as demonstrated by qPCR analysis (Additional file 1: Figure S3B).

**Fig. 3:**
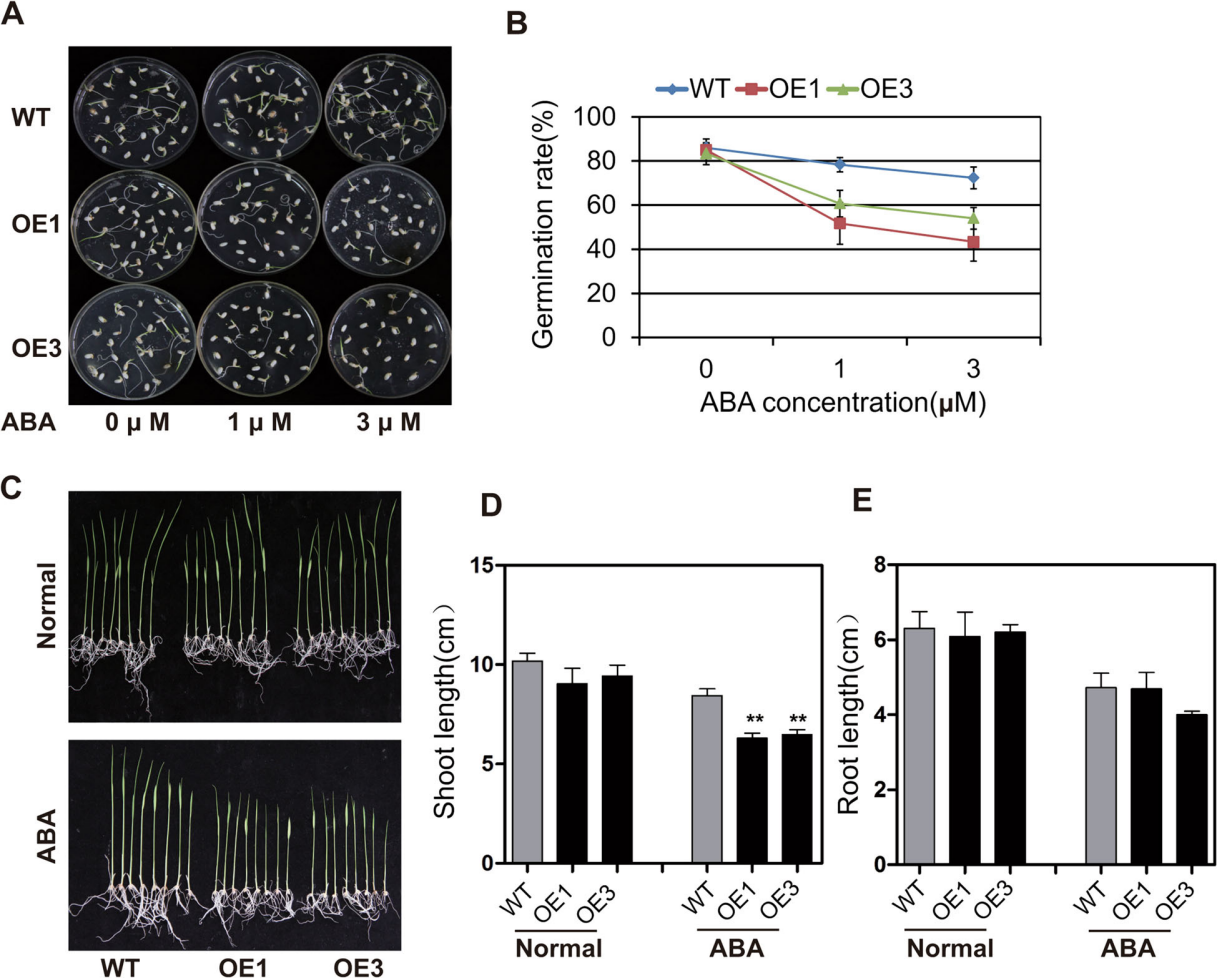
Increased ABA Sensitivity of *OsbZIP62V* transgenic plants at the germination and seedling stages. **a**, Germination performance of *OsbZIP62* overexpressors (OE1 and OE3) and WT seeds on 1/2-strength MS medium containing 0, 1, and 3 μM ABA at 7 days after initiation. **b**, Germination rate of transgenic *OsbZIP62* (OE1 and OE3) and the WT transgenic seeds on 1/2-strength MS medium containing 0, 1, and 3 μM ABA. The error bars indicate SEs based on three replicates. **c**, Performance of two independent *OsbZIP62V* transgenic lines (OE1 and OE3) and the WT on 1/2-strength MS medium containing 3 μM ABA. **d**, Shoot length of the transgenic lines and control lines on medium without or with 3 μM ABA. E, Root length of transgenic lines and control lines on medium without or with 3 μM ABA. Statistics of the length of the shoots and the roots at 7 days after growth. The data represent the means ± SE (*n* ≥ 8 each), **P < 0.01 according to Student’s *t* test

Although the germination rate of the *OsbZIP62V* overexpression transgenic lines (OE1 and OE3) was similar to that of the WT under 0 μM ABA, it was significantly lower than that of the WT under 1 and 3 μM ABA (Fig. 3a, b). Moreover, the shoot lengths of the *OsbZIP62V tr*ansgenic rice plants were significantly shorter than those of the WT rice plants under 3 μM ABA treatment (Fig. 3c-e). These results indicated that *OsbZIP62V* enhanced the sensitivity of the transgenic rice plants to ABA.

To further determine whether knocking out *OsbZIP62* could affect the sensitivity of transgenic plants to ABA, an *osbzip62* mutant was generated via the CRISPR/Cas9 method (Additional file 1: Figure S4). The results showed that the germination rate of the *osbzip62* mutant lines was almost the same as that of the wild-type Nipponbare plants under ABA treatment (Additional file 1: Figure S5). There was no significant difference in the length of the shoots and roots of *osbzip62* mutants and WT (data not shown). These results suggested that knocking out *OsbZIP62* did not significantly affect the ABA sensitivity of rice.

### *OsbZIP62V* improved the tolerance of transgenic rice to drought and oxidative stress

**Fig. 4:**
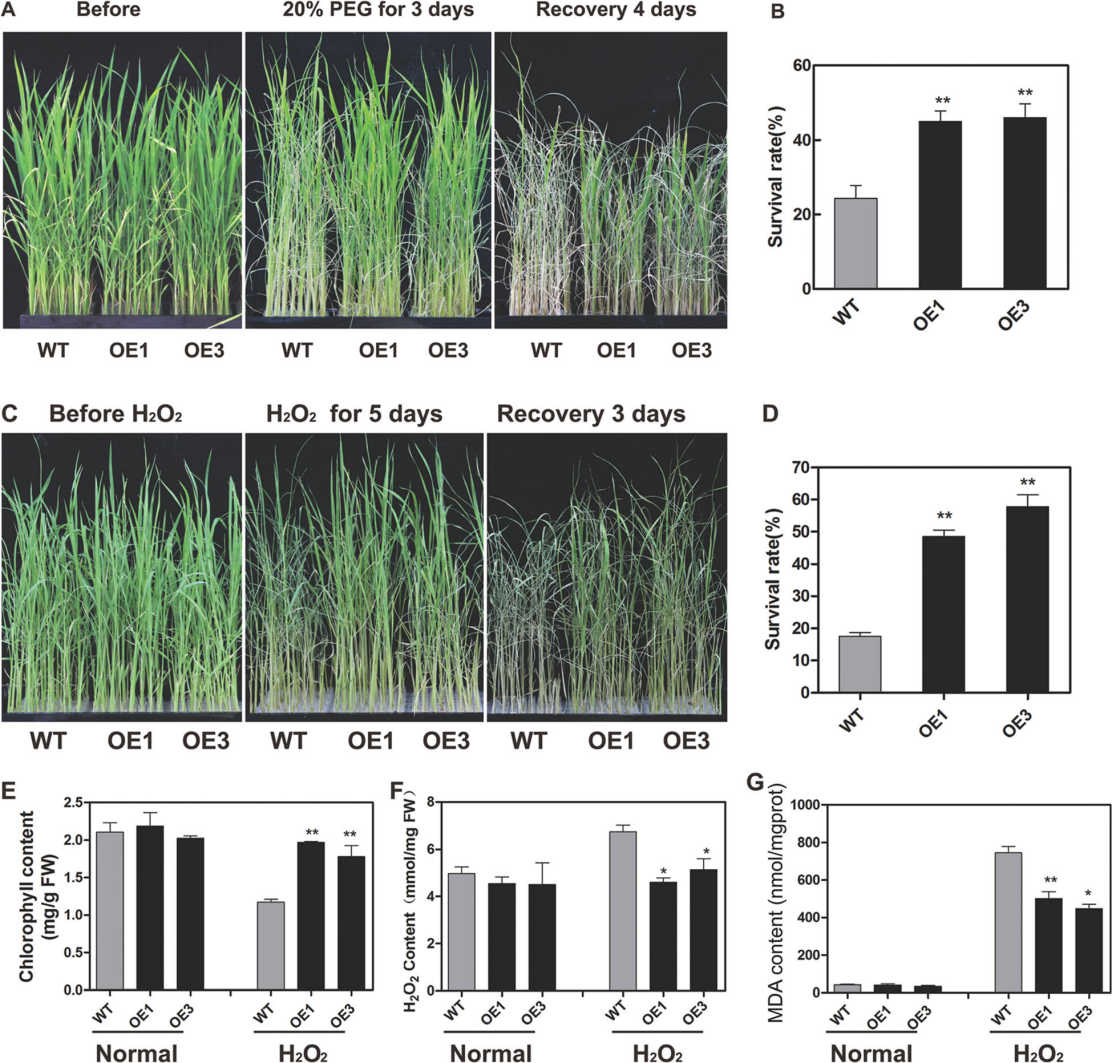
*OsbZIP62V* enhanced the tolerance of rice to drought and oxidative stress. **a**, *OsbZIP62V* transgenic rice plants and WT plants were cultured in 96-well plates and treated with 20% PEG for 3 days, after which they recovered for 4 days. **b**, Survival rate of *OsbZIP62V* transgenic plants and WT plants after PEG-simulated drought stress. The error bars indicate SEs based on three replicates, **P < 0.01 according to Student’s *t* test. **c**, Phenotype of *OsbZIP62V* under oxidative stress. *OsbZIP62V* lines and WT plants were cultured in 96-well plates and treated with 60 mM H_2_O_2_ for 5 days, after which they recovered for 3 days. **d**, Survival rate of *OsbZIP62V* transgenic plants and control plants after oxidative stress. The error bars indicate SEs based on three replicates. **e**, Total chlorophyll content in rice leaves. **f**, Total H_2_O_2_ content. **g**, Total MDA content. The data represent the means ± SDs (*n* = 4 or 5), *P < 0.05, **P < 0.01 according to Student’s *t* test. FW: fresh weight

To evaluate the putative biological function of *OsbZIP62*, we evaluated the drought tolerance of *OsbZIP62V* plants at the vegetative stage. Under PEG-simulated drought stress treatment, compared with the WT plants, the *OsbZIP62V* plants displayed less wilting (Fig. 4a). After recovery, 45–46% of the *OsbZIP62V* plants recovered, while the recovery rate of the WT plants was only 24% (Fig. 4b). The results indicated that *OsbZIP62* had a positive effect on rice osmotic stress tolerance.

In addition, the seedlings of the *OsbZIP62V* and WT plants were also treated with 60 mM H_2_O_2_ to determine their tolerance to oxidative stress. After treatment for 4–5 d, we found that the wilting degree of the overexpression transgenic plants was lower than that of the WT plants (Fig. 4c) and that the survival rate of the *OsbZIP62V* (48–55%) plants was significantly greater than that of the WT plants (18%) (Fig. 4d). Physiological analysis showed that, compared with the WT plants, the transgenic rice plants accumulated less H_2_O_2_ and malonaldehyde (MDA) and had greater amounts of chlorophyll (Fig. 4e-g). These results suggested that the activated expression of *OsbZIP62* improved the oxidative stress tolerance of the transgenic rice plants.

### Knocking out *OsbZIP62* decreased the tolerance of transgenic rice to drought and oxidative stress

**Fig. 5:**
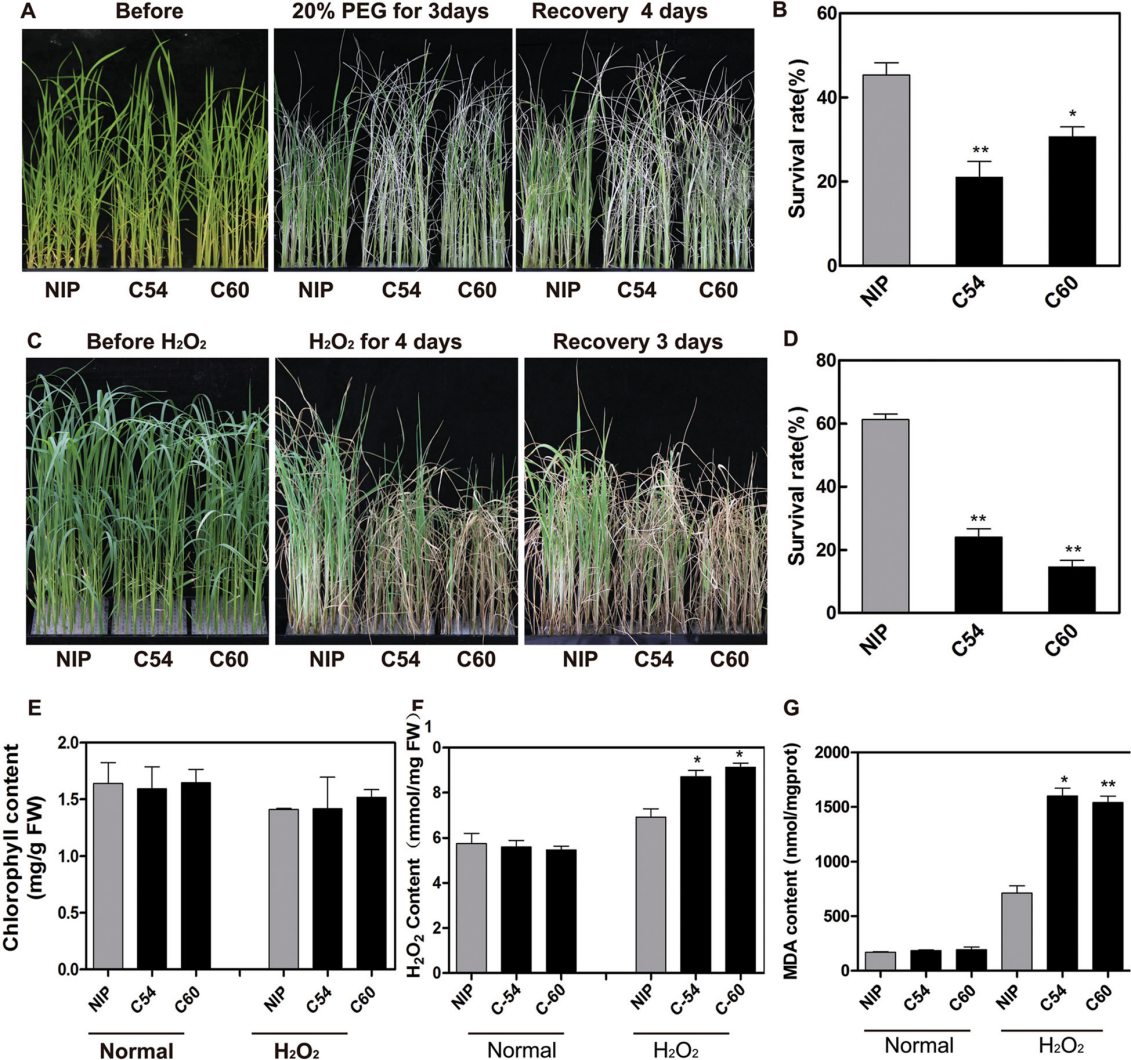
Decreased tolerance of the *osbzip62*-mutant plants to drought and oxidative stress. **a**, Phenotype of the *osbzip62*-mutant plants under PEG-simulated drought stress. *osbzip62* plants and WT plants were treated with 20% PEG for 3 days and then recovered for 4 days. **b**, Survival rate of *osbzip62* plants and WT plants after drought stress. The error bars indicate SEs based on three replicates. **c**, Phenotype of the *osbzip62* plants under oxidative stress. *Osbzip62* plants and WT plants were treated with 60 mM H_2_O_2_ for 4 days and then recovered for 3 days. **d**, Survival rate of *osbzip62* plants and WT plants after oxidative stress. The error bars indicate SEs based on three replicates. **e**, Total chlorophyll content in rice leaves. **f**, Total H_2_O_2_ content. **g**, Total MDA content. The data represent the means ± SDs (n = 4 or 5), *P < 0.05, **P < 0.01 according to Student’s *t* test. FW: fresh weight

To further validate the function of *OsbZIP62,* the tolerance of *osbzip62* mutants to drought and oxidative stress was evaluated. The leaf rolling of the *osbzip62-*mutant was significantly worse than that of the WT plants under PEG-simulated drought stress (Fig. 5a). The *osbzip62-*mutant had a significantly lower recovery (20–36%) than did the WT plants (45%) (Fig. 5b). Furthermore, the degree of wilting of the *osbzip62* mutants was higher than that of the WT plants under H_2_O_2_ treatment (Fig. 5c). After recovery, the survival rate of the *osbzip62* mutants were only 14–24%, while that of the WT was 61% (Fig. 5d). Physiological analysis showed that the contents of H_2_O_2_ and MDA in the *osbzip62* mutant were significantly greater than that in the WT (Fig. 5e-g). These results demonstrate that knocking out *OsbZIP62* reduces rice tolerance to drought and oxidative stress.

### Identification of putative target genes regulated by OsbZIP62

**Fig. 6:**
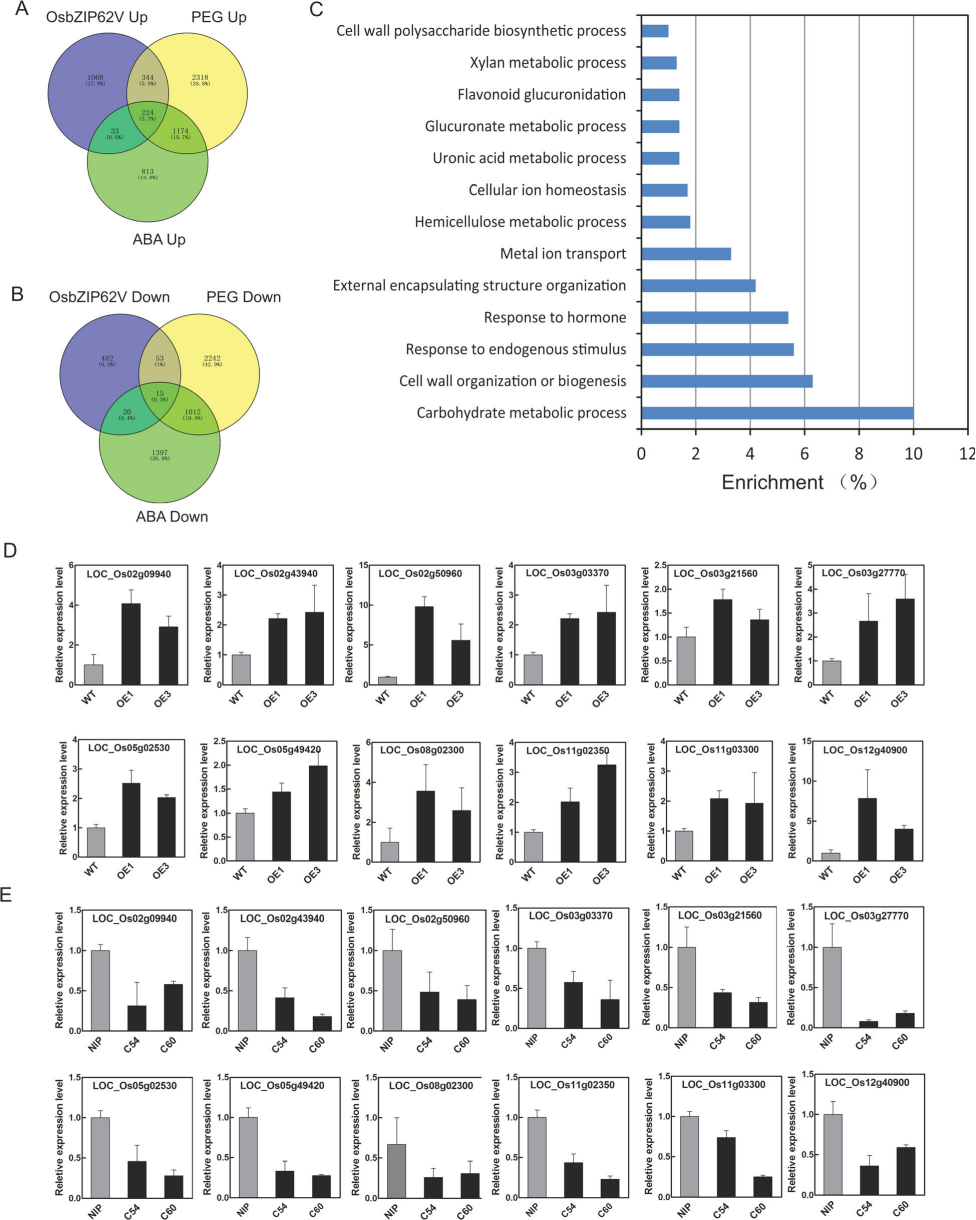
Transcriptome analysis of *OsbZIP62V* plants, *osbzip62*-mutant plants and WT plants. **a**, Venn diagram for the numbers of upregulated genes in *OsbZIP62V* plants, PEG-upregulated genes, and ABA-upregulated genes. **b**, Venn diagram of the numbers of downregulated genes in *OsbZIP62V* plants, PEG-downregulated genes, and ABA-downregulated genes. **c**, Classification of partially upregulated genes in the *OsbZIP62V* transgenic plants compared with the WT plants. **d**, Relative expression level of DEGs in *OsbZIP62V* and WT plants. **e**, Relative expression level of DEGs in *osbzip62* plants and WT plants. The date represent the means ± SE (*n* = 3)

To elucidate the molecular mechanism of *OsbZIP62* in rice drought tolerance, the transcriptomes of *OsbZIP62V*, *osbzip62* and WT plants were analyzed using high-throughput sequencing. Differentially expressed genes (DEGs) between the transgenic rice plants and WT plants were analyzed. There were 1669 upregulated genes (fold-change ≥2.0) and 570 downregulated genes (fold change ≤0.5) in the *OsbZIP62V* transgenic plants compared with WT plants (Additional file 2: Table S1). In the *osbzip62* mutants, 663 genes are upregulated, and 480 genes were downregulated (Additional file 3: Table S2). Among the upregulated genes in the *OsbZIP62V* transgenic plants, 568 genes were also induced by PEG-simulated drought stress, and 257 genes were induced by ABA treatment (the RNA-seq of Nipponbare plants under PEG and ABA treatment was performed by our lab, and these data have not been published), which suggests that these genes upregulated by *OsbZIP62* may participate in drought and ABA responses (Fig. 6a). The enriched upregulated genes in the *OsbZIP62V* plants mainly belong to the following biological process categories: carbohydrate metabolic process, response to abiotic stimuli, cell wall organization or biogenesis, response to hormones, and cellular ion homeostasis (Fig. 6c). The Expression levels of several DEGs were checked by qPCR. The results confirmed that the expression of most of the selected DEGs (e.g., *LOC_Os02g43940*, *LOC_Os05g49420*, *LOC_Os11g03300* and *LOC_Os03g03370*) was greater in *OsbZIP62V* overexpression transgenic lines and lower in *osbzip62* mutants than in WT plants (Fig. 6d and e). These results demonstrated that the transcription of these DEGs was affected by *OsbZIP62*, suggesting that these DEGs might be target genes regulated by *OsbZIP62*.

We also analyzed the DEGs whose expression tended to change in the opposite manner in *OsbZIP62V* plants and *osbzip62* mutants and found 85 DEGs were upregulated in the *OsbZIP62V* plants but downregulated in the *osbzip62* mutants (Additional file 4: Table S3). These DEGs encode different functional proteins, such as transcription factors, protease inhibitors, and antioxidant enzymes (e.g., *LOC_Os07g01370*, *LOC_Os02g09940* and *LOC_Os05g02530* encode a peroxidase; *LOC_Os03g20760* and *LOC_Os11g02350* encode a protease inhibitor; *LOC_Os08g02300* and *LOC_Os12g43110* encode a transcription factor; *LOC_Os02g56920* encodes a wax synthesis protein; and *LOC_Os03g21560* encodes a photosystem II repair protein).

**Fig. 7:**
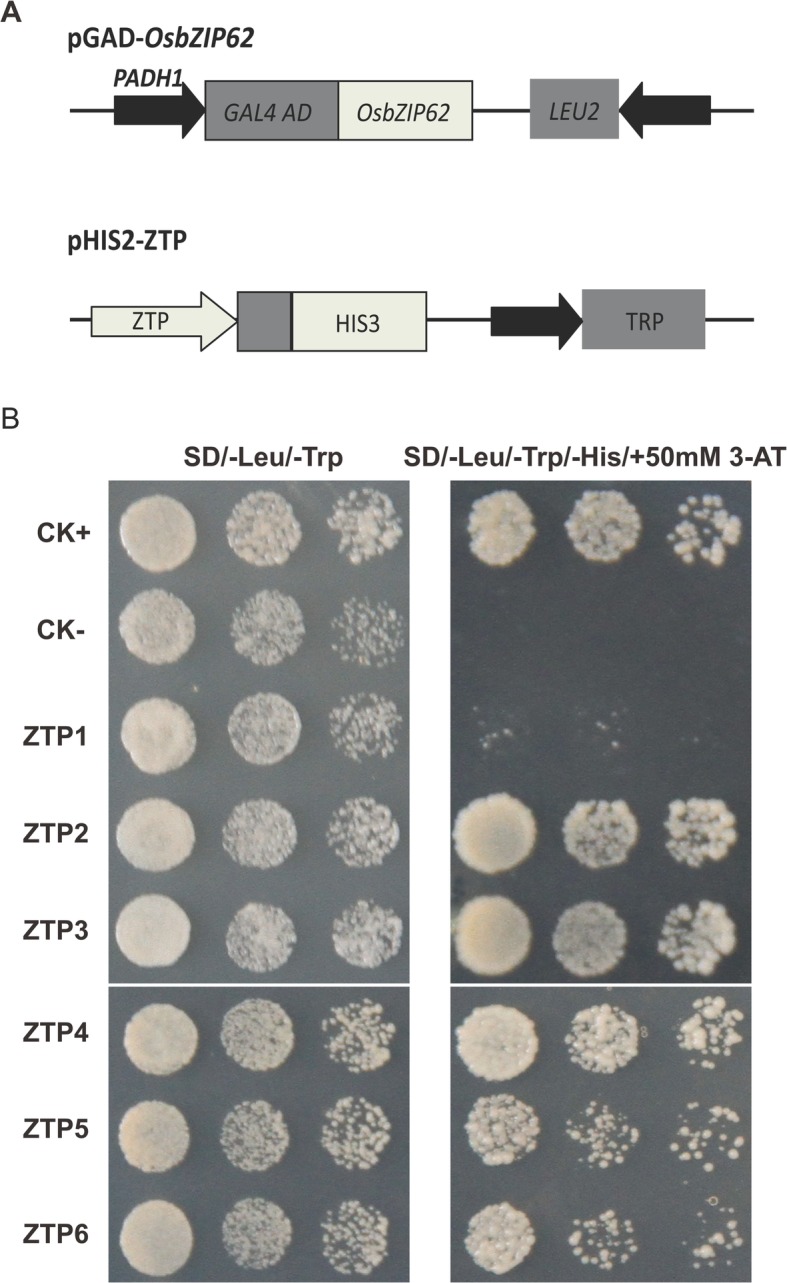
Identification of DNA binding activity of OsbZIP62. **a**, Schematic structure of the yeast one-hybrid expression construct pGAD-OsbZIP62 and the reporter construct pHIS2.1-ZTP (OsbZIP62 putative target gene promoter). **b**, Growth performance of transformants on SD/−Leu−/Trp/−His medium containing 30 mM 3-AT. ZTP1-ZTP6 indicates the pGAD-OsbZIP62 plus pHIS2.1-cis (with the promoters of *LOC_Os11g02350*, *LOC_Os02g56920*, *LOC_Os03g21560*, *LOC_Os07g01370*, *LOC_Os11g03300* and *LOC_Os03g03370* in pHIS2.1, respectively). ck+: positive control, ck-: negative control (pGADT7-rec2-OsbZIP62 plus p53HIS2)

To further identify the target genes of *OsbZIP62*, we first analyzed the promoters of the DEGs and identified promoters of several DEGs (e.g., *LOC_Os03g03370* (*DSM2*), *LOC_Os11g03300* (*OsNAC10*), and *LOC_Os02g56920* (*OsGL1*)) containing ABREs or G-box cis-elements that may bind bZIP transcription factors (data not shown). We investigated whether the OsbZIP62 protein could bind to the promoters of these putative target genes through a yeast one-hybrid assay. A pGAD-OsbZIP62 plasmid (containing the OsbZIP62 putative DNA domain fused to the GAL4 active domain) and pHIS-cis reporter construct (~ 1 kb promoters of six presumed target genes) were co-transformed into yeast strain Y187 (Fig. 7a). As indicated by the activation of the reporter genes, OsbZIP62 could bind to the promoters of several genes (i.e., *LOC_Os02g56920*, *LOC_Os03g21560*, *LOC_Os07g01370*, *LOC_Os11g03300*, and *LOC_Os03g03370*) (Fig. 7b). These results indicated that OsbZIP62 exhibits DNA binding activity and might directly regulate the expression of these target genes.

### OsbZIP62 interacts with stress/ABA-activated protein kinases and might be activated by phosphorylation

**Fig. 8:**
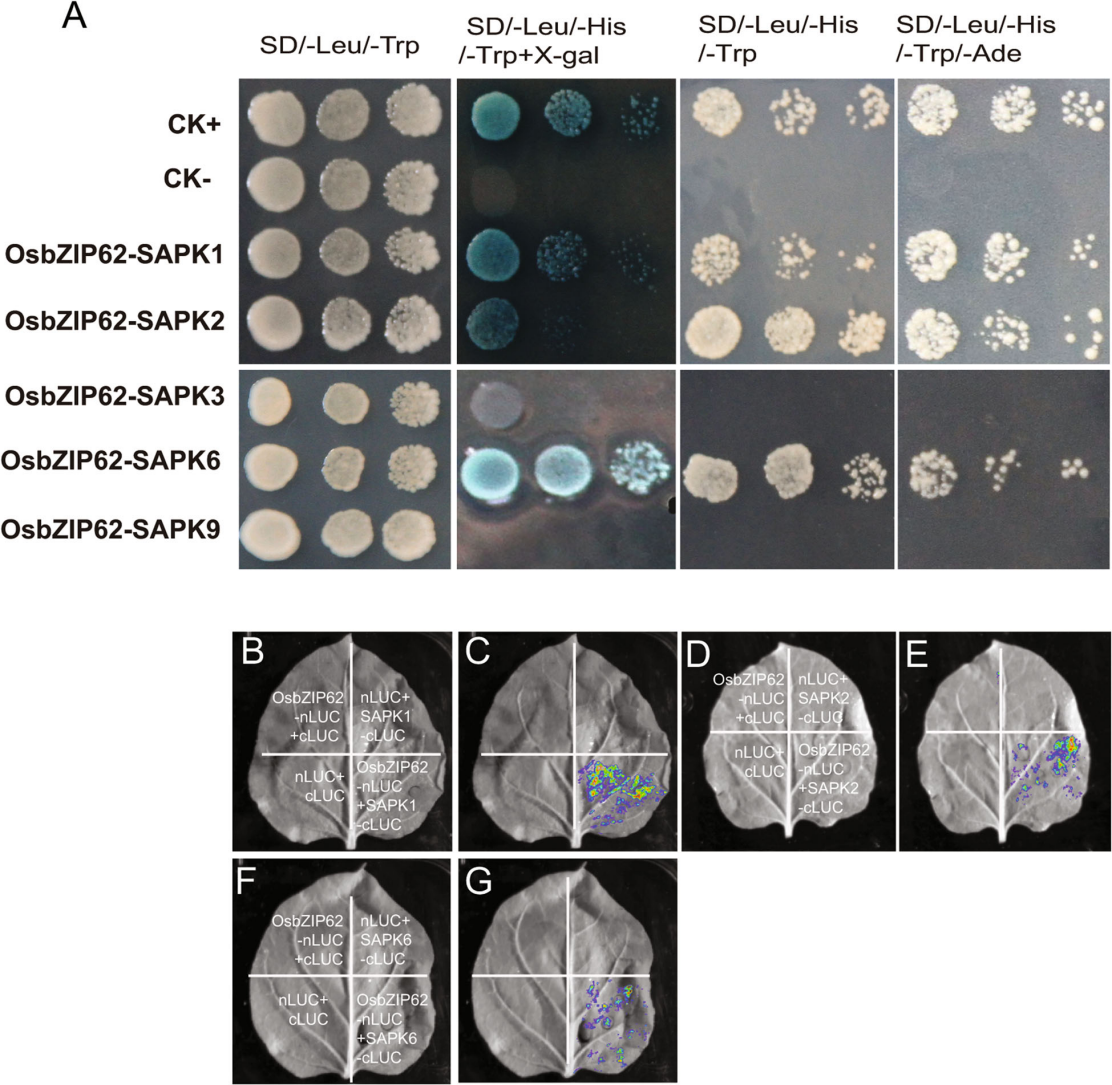
OsbZIP62 interacts with SnRK2 protein kinases. **a**, Yeast two-hybrid assays of OsbZIP62 and SAPK members. SAPK1/SAPK2/SAPK3/SAPK6/SAPK9 were separately fused to the pGADT7 activation domain, which were then transformed into Y2H Gold cells via pGBKT7-OsbZIP62. CK+ and CK- indicate positive and negative controls, respectively. **b**-**g**, Confirmation of the in vivo interactions of OsbZIP62 with SAPK1/SAPK2/SAPK6 via Luciferase complementation imaging assays in tobacco leaves

The whole length of OsbZIP62 had no transcriptional activation activity, whereas the N-terminus of OsbZIP62 displayed transactivation activity, even when C-terminus (69–274 amino acids) was deleted. Therefore, we proposed that OsbZIP62 might also interact with SAPK protein kinases and be phosphorylated. To test this hypothesis, using a yeast two-hybrid assay, we first investigated the interaction between OsbZIP62 and five SAPK family members from rice. The results showed that OsbZIP62 could interact with SAPK1, SAPK2 and SAPK6 (Fig. 8a). The interaction between OsbZIP62 and the three SAPKs was further confirmed by luciferase complementation imaging assays in tobacco leaves (Fig. 8b-g). In summary, these results indicated that OsbZIP62 interacted with several SAPKs, suggesting that OsbZIP62 might be activated by the phosphorylation of these protein kinases.

## Discussion

### *OsbZIP62* encodes stress-responsive bZIP transcription factors and positively regulates rice drought tolerance and oxidative tolerance

Transcription factors play an important role in regulating the stress response in plants. In Arabidopsis, many bZIP transcription factors are involved in the ABA-mediated stress response, such as *ABI5* [16, 29], *ABF1/3/4*, and *ABRE3* [20, 30–32]. However, only a few of them have been reported in rice, such as *OsbZIP*23, *OsbZIP*46, *OsbZIP*72 [23–25, 27, 33], and the biological functions of many other bZIP proteins remain unclear. Here, we report a novel rice bZIP transcription factor, *OsbZIP*62, that is involved in rice stress responses. Phylogenetic analysis showed that *OsbZIP62* belongs to the third subfamily of bZIP family (Additional file 1: Figure S2). Previous studies have showed that some members of the bZIP third subfamily are also induced by abiotic stress and are involved in drought and ABA signalling pathway [24]. *OsbZIP62* was obviously induced by various abiotic stresses (i.e., PEG, H_2_O_2_, salt, and heat) and hormone (i.e., ABA) treatments (Fig. 1a). In addition, there are many predicted stress-related cis*-*elements, including ABREs and HSEs, within the *OsbZIP62* promoter region. (Fig. 1b). The current study showed that *OsbZIP62V* significantly enhanced the tolerance to drought and oxidative stress (Fig. 4), but compared with WT plants, the *osbzip62* mutants displayed reduced drought stress tolerance (Fig. 5). In addition, the expression of hundreds of drought-responsive genes was upregulated in *OsbZIP62V* plants (Fig. 6 and Additional file 2: Table S1). These findings clearly demonstrated that *OsbZIP62* encodes a novel bZIP transcription factor that positively regulates the drought tolerance and oxidative tolerance of rice.

### *OsbZIP62* might participate in the ABA response

It has been shown that bZIP transcription factors constitute the key component of ABA signalling. For example, many members of the group C bZIP transcription factors, which are known as the ABF/AREB/ABI5 subfamily, participate in ABA signal transduction of and the stress response. It is well known that SnRK2 phosphorylation of ABF/AREB is an important part of the complete ABA signalling pathway in Arabidopsis [34, 35]. In particular, TRAB1, which is homologous to OsbZIP62, has been determined to be phosphorylated by SAPKs [36, 37]. In this study, it was found that OsbZIP62 interacted with the SAPK1/2/6 ABA signalling components (Fig. 8), suggesting that OsbZIP62 may be regulated by these protein kinase phosphorylation pathways. Furthermore, we found that the full-length OsbZIP62 protein showed no transcriptional activity but that the N-terminal sequence (amino acids 1–68) exhibited transactivation activity in yeast (Fig. 2a). These results are similar to those of OsbZIP46 and suggest that the transactivation activity of OsbZIP62 might be activated through phosphorylation by these protein kinases; however, the details need to be further studied.

In this study, the activation of OsbZIP62 increased rice sensitivity to ABA (Fig. 3). Furthermore, the expression of a number of ABA-responsive genes was upregulated in *OsbZIP62V* plants (Fig. 6a). On the other hand, knocking out *osbzip62* did not alter the sensitivity to ABA (Additional file 1: Figure S5), possibly because of the abundant bZIP proteins involved in ABA signalling. Nevertherless, all of these findings supported that the transcription and post-translation of OsbZIP62 is regulated by ABA signalling and that OsbZIP62 also partially participates in ABA signalling by regulating many ABA-responsive genes.

### *OsbZIP62* regulates the expression of many stress-related genes

Transcription factors and cis-elements are the core components of plant regulatory networks. Identification of direct downstream target genes is an effective strategy in terms of clarification of the function of the OsbZIP62 transcription factor. Not surprisingly, OsbZIP62 altered the expression levels of many genes. The expression of nearly more than 1000 genes was upregulated more than 2-fold in the *OsbZIP62V* overexpressors plants (Additional file 2: Table S1). On the other hand, the mutant of OsbZIP62 also exhibited expression changes of hundreds of genes (Additional file 3: Table S2). Among the DEGs upregulated in *OsbZIP62V*, more than one-third of them were drought responsive, which is approximately 14% of total drought responsive genes (Fig. 6a). Many genes that are upregulated in *OsbZIP62V* encode reported or predicted proteins involved in stress resistance, such as transcription factors (*LOC_Os11g03300* (*OsNAC10*)), β-carotene hydroxylase (*LOC_Os03g03370* (*DSM2*)), protease inhibitors (*LOC_Os11g02350*), and wax synthesis proteins (*LOC_Os02g56920* (*OsGL1*)). *OsGL1* has been reported to be one of the genes controlling wax synthesis; this gene is involved in wax accumulation in leaf cuticles and directly affects the drought resistance of rice [38, 39]. *OsNAC10*, an NAC transcription factor, enhances drought resistance and increases the yield of rice under drought conditions [40]. *DSM2* enhances the resistance of rice to drought and oxidative stress by increasing the biosynthesis of lutein and ABA [41]. Yeast one-hybrid assays further confirmed that OsbZIP62 could bind to the promoters of these genes (e.g., *OsGL1*, *OsNAC10*, and *DSM2*) (Fig. 7). These findings suggest that the induced expression of 14% drought stress-related genes by *OsbZIP62* contributes to the enhanced drought tolerance of the transgenic rice plants.

## Conclusions

We screened drought tolerance candidate genes via a transcriptome analysis of several typical rice varieties with different drought tolerances and selected a novel bZIP gene, *OsbZIP62*, as one of the candidate genes. *OsbZIP62* belongs to the third subfamily of bZIP transcription factors in rice. The expression of *OsbZIP62* was obviously induced by drought, heat, salt, H_2_O_2_ and ABA treatment. By modulating many stress-related genes, *OsbZIP62* is a positive regulator of ABA signalling, drought, and oxidative stress responses. The OsbZIP62 protein localized to the nucleus, and its function in activating downstream genes may depend on posttranslational modification by SAPKs. Thus, we concluded that *OsbZIP62* encodes a novel stress-responsive bZIP transcription factor and positively regulates the rice drought and oxidative stress responses.

## Methods

### Construction of transgenic materials

The full-length cDNA of *OsbZIP62* was amplified from the cDNA of Nipponbare (*Oryza sativa L. japonica*. cv. Nipponbare) and then cloned into a pEASY-Blunt simple cloning vector for sequencing. The seeds of Nipponbare were obtained from the germplasm bank in our lab. The seeds of transgenic japonica rice Kitaake with *OsbZIP62-VP64* were acquired from Fujian Agriculture and Forestry University, and these transgenic rice plants were generated as described previously [42].

CRISPR constructs were designed according to a previously described protocol [43, 44], and the vectors were transformed into Nipponbare using the *Agrobacterium*-mediated method. The primers used in this study are listed in Additional file 5: Table S4. Individual T0 transformants were analyzed by sequencing their OsbZIP62 target regions, which were amplified by PCR. Mutations were detected by aligning the sequence chromatograms of these PCR products with those of the WT controls. Several individual homozygous or heterozygous plants were obtained, and two homozygous lines, C54 and C60, were selected for further experiments.

### Plant materials, growth conditions and stress treatments

To determine the gene expression profiles, we planted the Nipponbare plants in culture solution in a greenhouse with a 16-h light/8-h dark cycle. Rice seedlings at the four-leaf stage were treated with hormones or subjected to non-biological stresses such as cold stress (rice seedlings were transferred to a growth chamber at 4 °C), heat stress (rice seedlings were exposed to a 42 °C growth chamber), osmotic stress (15% (m/v) PEG6000), oxidative stress (1% H_2_O_2_) and salt stress (150 mM NaCl) and then sampled at 0, 1, 2, 4, 8, 12 and 24 h. For the phytohormone ABA treatment, 0.1 mM ABA was added to the culture medium of the rice plants.

To determine the ABA sensitivity of the transgenic plants at the seedling stage, positive transgenic seeds were selected in one-half-strength MS culture medium supplemented with 50 mg/L hygromycin. WT control lines were germinated on 1/2-strength MS medium. Seedlings (12 plants each, 3 repeats) were transplanted to 1/2-strength MS culture medium or 1/2-strength MS medium containing 3 μM ABA and were grown for 7 days. To testing the ABA sensitivity of transgenic plants at the germination stage, the seeds of positive transgenic plants and wild type plants (approximately 30 seeds, three repetitions) were germinated on 1/2-strength MS medium containing a concentration gradient of ABA (0, 1, 3, and 6 μM), and the germination rate of the seeds was calculated after 7 days.

### Determination of physiological indices related to stress

For all stresses, the seeds of T3 lines were germinated on 1/2-strength MS medium supplemented with 50 mg/L hygromycin, and the seeds of WT also grown on 1/2-strength MS medium. At the four-leaf stage, osmotic stress (20% PEG treatment for 3 days before rewatering) and oxidative stress (60 mM H_2_O_2_ treatment for 4–5 days) were applied, and the survival rates were measured as described previously [45, 46]. Moreover, we performed a biochemical analysis of reactive oxygen species (ROS). H_2_O_2_ was extracted from the leaves, and its contents were determined following the manufacturer’s instructions as described previously [47]. The activity of ROS-scavenging enzymes, soluble protein and MDA contents were determined according to the manufacturer’s instructions (Nanjing Institute of Bioengineering, Jiangsu, China). The chlorophyll contents were measured as described previously [48].

### RNA isolation and quantification of gene expression

Total RNA was extracted from the rice leaves using TRNzol-A^+^ reagent (Tiangen) according to the manufacturer’s instructions. First-strand cDNA was synthesized via EasyScript One-Step gDNA Removal and cDNA Synthesis Super Mix (Transgen). Real-time quantitative PCR (qPCR) was performed on an optical 96-well plate with a Bio-Rad CFX96 Real-Time PCR Detection System using TransStart Green qPCR SuperMix (Transgen). The PCR procedure was as follows: 94 °C for 30 s, followed by 40 cycles at 94 °C for 5 s, 55 °C for 15 s and 72 °C for 10 s. The rice *OsAct8* gene (No. AY212324) was used as an internal control to normalize the target gene expression, and relative expression levels were determined as described previously [49].

### RNA sequencing

Three biological replicates (30 plants each) of wild-type, *OsbZIP62V* and *osbzip62* mutant plants were sampled for RNA sequencing. The total RNA was extracted using the Trizol Reagent (Life Technologies). The qualified RNA samples were then used for library construction following the specifications of the TruSeq RNA Sample Preparation v2 Guide (Illumina), and RNA sequencing was conducted with Illumina Hiseq 2500 at Shanghai Personal Biotechnology Co., Ltd. (Shanghai, China). We used SeqPrep to strip adaptors and/or merge paired reads that overlapped into single reads and used Sickle to remove low-quality reads. The clean data were then aligned to the reference genome of rice using HISAT2 v2.1.0. FPKM (fragments per kilobase per millon mapped reads) was then calculated to estimate the expression level of the genes. DESeq2 v1.6.3 was used to analyse the differential gene expression between two samples, and genes with q<0.05 and |log2_ratio|>1 were identified as differentially expressed genes. GO (Gene Ontology; http://geneontology.org/) enrichment of DEGs was implemented by hypergeometric tests in which *p*-value is calculated and adjusted as q-value. GO terms with q < 0.05 were considered significantly enriched.

### Subcellular localization

To study the subcellular localization of OsbZIP62 protein, the whole length of OsbZIP62 was cloned into a pCAMBIA1300GFP plant expression vector after digestion of XbaI and BamHI enzyme; thus, OsbZIP62 was fused to GFP. Rice protoplasts were subsequently prepared from rice etiolated shoots, transformed with GFP or OsbZIP62-GFP constructs, and subjected to PEG treatment. GFP fluorescence was detected under an Olympus confocal microscope 24 h after transformation.

### Transactivation activity assay in yeast

The transcriptional activation activity of OsbZIP62 was studied in yeast cells. The full length or truncated fragments of *OsbZIP62* were fused in frame with the receptor GAL4 DNA-binding domain in a pGBKT7 vector by recombination reactions. These constructs were separately transformed into Y2H Gold cells. The transformants were evaluated by serial dilution on SD/−Trp/−His medium containing different concentrations of 3-amino-1, 2, 4-triazole (3-AT) or X-α-Gal.

### Yeast one-hybrid assays

We selected six genes (i.e., *LOC_Os11g02350*, *LOC_Os02g56920*, *LOC_Os03g21560*, *LOC_Os07g01370*, *LOC_Os11g03300*, and *LOC_Os03g03370*) that were upregulated in *OsbZIP62V* plants and downregulated in *osbzip62* mutants. The upstream approximately 1 kb long promoter of these potential target genes was cloned from the genomic DNA of Nipponbare. For the yeast one-hybrid assays, the promoter sequences of these genes were cloned into the yeast expression vector pHIS2.1 after digestion of the SmaI and EcoRI enzymes and then was co-transformed with the pGAD-T7-OsbZIP62 into Y187 yeast cells. The DNA-protein interaction was evaluated by transformant growth assays on SD/−Leu/−Trp/−His plates supplied with 50 mM of 3-AT.

### Yeast two-hybrid assays

Yeast two-hybrid assays were carried out with a Matchmaker™ Gold Yeast Two-Hybrid System (Clontech). The coding regions of OsbZIP62 and SAPKs were cloned into pGBKT7 and pGAD-T7 vectors to generate bait and prey vectors, respectively. These vectors were subsequently co-transformed into the Y2H Gold strain (Clontech). Protein interactions were evaluated by transformant growth assays on SD/−Leu/−Trp/−His, SD/−Leu/−Trp/−His with X-Gal, and SD/− Leu/−Trp/−His-Ade at 30 °C for 3–5 days.

### LUC complementary imaging assays

OsbZIP62 and SAPK1/SAPK2/SAPK6 were cloned into the pCAMBIA1300-nLUC and pCAMBIA1300-cLUC firefly luciferase vectors, respectively. All of the constructs were introduced into *A. tumefaciens* strain EHA105, which were then injected into 3-week-old tobacco leaves to transiently transform the tobacco epidermal cells [50]. The fluorescence signal was detected 3 days after infiltration under a plant in vivo imaging system (NightSHADE LB 985, Berthold).

## Additional files

Additional file 1: **Figure S1.** Protein sequence alignment of OsbZIP62 and the third subfamily bZIP transcription factors in rice. **Figure S2.** Molecular phylogenetic tree of the third bZIP subfamily members. **Figure S3.** Identification of *OsbZIP62-VP64* overexpression lines. **Figure S4.** Characterization of *osbzip62* mutants. **Figure S5.** ABA sensitivity of *osbzip62* mutants. (DOCX 2016 kb)

Additional file 2: **Table S1.** DEGs in *OsbZIP62-VP64* overexpressor (XLSX 177 kb)

Additional file 3: **Table S2.** DEGs in *osbzip62* mutant (XLSX 96 kb)

Additional file 4: **Table S3.** Genes proposed to be regulated by OsbZIP62 (XLSX 14 kb)

Additional file 5: **Table S4.** List of primers used in this study (XLSX 14 kb)

## Data Availability

The RNA-seq data supporting the results of this article have been submitted to the GEO at NCBI with the accession number GSE122887.

## Abbreviations

ABA: abscisic acid
bZIP: basic leucine zipper motif
CAT: catalase
DEGs: differentially expressed genes
MDA: malonaldehyde
MS: murashige and skoog
PEG: polyethylene glycol
SAPK: stress/ABA activated protein kinases
SOD: superoxide dismutase

## References

[1] Rodriguez-Uribe L, O'Connell MA (2006). A root-specific bZIP transcription factor is responsive to water deficit stress in tepary bean (Phaseolus acutifolius) and common bean (P. vulgaris). J Exp Bot.

[2] Zhang Q (2007). Strategies for developing green super Rice. Proc Natl Acad Sci U S A.

[3] Luo LJ (2010). Breeding for water-saving and drought-resistance rice (WDR) in China. J Exp Bot.

[4] Ouyang SQ, Liu YF, Liu P, Lei G, He SJ, Ma B, Zhang WK, Zhang JS, Chen SY (2010). Receptor-like kinase OsSIK1 improves drought and salt stress tolerance in rice (Oryza sativa) plants. Plant J.

[5] Verma V, Ravindran P, Kumar PP (2016). Plant hormone-mediated regulation of stress responses. BMC Plant Biol.

[6] Seif El-Yazal SA, Seif El-Yazal MA, Dwidar EF, Rady MM (2015). Phytohormone crosstalk research: cytokinin and its crosstalk with other phytohormones. Curr Protein Pept Sci.

[7] Peleg Z, Blumwald E (2011). Hormone balance and abiotic stress tolerance in crop plants. Curr Opin Plant Biol.

[8] Park E, Kim TH (2017). Production of ABA responses requires both the nuclear and cytoplasmic functional involvement of PYR1. Biochem Biophys Res Commun.

[9] Nishimura N, Sarkeshik A, Nito K, Park SY, Wang A, Carvalho PC, Lee S, Caddell DF, Cutler SR, Chory J (2010). PYR/PYL/RCAR family members are major in-vivo ABI1 protein phosphatase 2C-interacting proteins in Arabidopsis. Plant J.

[10] Hubbard KE, Nishimura N, Hitomi K, Getzoff ED, Schroeder JI (2010). Early abscisic acid signal transduction mechanisms: newly discovered components and newly emerging questions. Genes Dev.

[11] Zhang L, Xia C, Zhao G, Liu J, Jia J, Kong X (2015). A novel wheat bZIP transcription factor, TabZIP60, confers multiple abiotic stress tolerances in transgenic Arabidopsis. Physiol Plant.

[12] Kang JY, Choi HI, Im MY, Kim SY (2002). Arabidopsis basic leucine zipper proteins that mediate stress-responsive abscisic acid signaling. Plant Cell.

[13] Hossain MA, Cho JI, Han M, Ahn CH, Jeon JS, An G, Park PB (2010). The ABRE-binding bZIP transcription factor OsABF2 is a positive regulator of abiotic stress and ABA signaling in rice. J Plant Physiol.

[14] Jakoby M, Weisshaar B, Droge-Laser W, Vicente-Carbajosa J, Tiedemann J, Kroj T, Parcy F (2002). bZIP transcription factors in Arabidopsis. Trends Plant Sci.

[15] Matiolli C. C., Tomaz J. P., Duarte G. T., Prado F. M., Del Bem L. E. V., Silveira A. B., Gauer L., Correa L. G. G., Drumond R. D., Viana A. J. C., Di Mascio P., Meyer C., Vincentz M. (2011). The Arabidopsis bZIP Gene AtbZIP63 Is a Sensitive Integrator of Transient Abscisic Acid and Glucose Signals. PLANT PHYSIOLOGY.

[16] Bensmihen S, Giraudat J, Parcy F (2005). Characterization of three homologous basic leucine zipper transcription factors (bZIP) of the ABI5 family during Arabidopsis thaliana embryo maturation. J Exp Bot.

[17] Wang B, Li C, Kong X, Li Y, Liu Z, Wang J, Li X, Yang Y (2018). AtARRE, an E3 ubiquitin ligase, negatively regulates ABA signaling in Arabidopsis thaliana. Plant Cell Rep.

[18] Gao S, Gao J, Zhu X, Song Y, Li Z, Ren G, Zhou X, Kuai B (2016). ABF2, ABF3, and ABF4 promote ABA-mediated chlorophyll degradation and leaf senescence by transcriptional activation of chlorophyll catabolic genes and senescence-associated genes in Arabidopsis. Mol Plant.

[19] Fujita Y, Fujita M, Satoh R, Maruyama K, Parvez MM, Seki M, Hiratsu K, Ohme-Takagi M, Shinozaki K, Yamaguchi-Shinozaki K (2005). AREB1 is a transcription activator of novel ABRE-dependent ABA signaling that enhances drought stress tolerance in Arabidopsis. Plant Cell.

[20] Kim S, Kang JY, Cho DI, Park JH, Kim SY (2004). ABF2, an ABRE-binding bZIP factor, is an essential component of glucose signaling and its overexpression affects multiple stress tolerance. Plant J.

[21] Zhao Y, Zhao S, Mao T, Qu X, Cao W, Zhang L, Zhang W, He L, Li S, Ren S (2011). The plant-specific actin binding protein SCAB1 stabilizes actin filaments and regulates stomatal movement in Arabidopsis. Plant Cell.

[22] Nijhawan A, Jain M, Tyagi AK, Khurana JP (2008). Genomic survey and gene expression analysis of the basic leucine zipper transcription factor family in rice. Plant Physiol.

[23] Xiang Y, Tang N, Du H, Ye H, Xiong L (2008). Characterization of OsbZIP23 as a key player of the basic leucine zipper transcription factor family for conferring abscisic acid sensitivity and salinity and drought tolerance in rice. Plant Physiol.

[24] Lu G, Gao C, Zheng X, Han B (2009). Identification of OsbZIP72 as a positive regulator of ABA response and drought tolerance in rice. Planta.

[25] Tang N, Zhang H, Li X, Xiao J, Xiong L (2012). Constitutive activation of transcription factor OsbZIP46 improves drought tolerance in rice. Plant Physiol.

[26] Gui J, Zheng S, Liu C, Shen J, Li J, Li L (2016). OsREM4.1 interacts with OsSERK1 to coordinate the interlinking between abscisic acid and Brassinosteroid signaling in Rice. Dev Cell.

[27] Tang N, Ma S, Zong W, Yang N, Lv Y, Yan C, Guo Z, Li J, Li X, Xiang Y, et al. MODD mediates deactivation and degradation of OsbZIP46 to negatively regulate ABA signaling and drought resistance in rice. Plant Cell. 2016. 10.1105/tpc.16.00171 PMC5059794 27468891

[28] Qiu J, Hou Y, Wang Y, Li Z, Zhao J, Tong X, Lin H, Wei X, Ao H, Zhang J (2017). A Comprehensive Proteomic Survey of ABA-Induced Protein Phosphorylation in Rice (*Oryza sativa* L.). Int J Mol Sci.

[29] Carles C, Bies-Etheve N, Aspart L, Leon-Kloosterziel KM, Koornneef M, Echeverria M, Delseny M (2002). Regulation of Arabidopsis thaliana Em genes: role of ABI5. Plant J.

[30] Uno Y, Furihata T, Abe H, Yoshida R, Shinozaki K, Yamaguchi-Shinozaki K (2000). Arabidopsis basic leucine zipper transcription factors involved in an abscisic acid-dependent signal transduction pathway under drought and high-salinity conditions. Proc Natl Acad Sci U S A.

[31] Fernando VCD, Al Khateeb W, Belmonte MF, Schroeder DF (2018). Role of Arabidopsis ABF1/3/4 during det1 germination in salt and osmotic stress conditions. Plant Mol Biol.

[32] Yoshida T, Fujita Y, Sayama H, Kidokoro S, Maruyama K, Mizoi J, Shinozaki K, Yamaguchi-Shinozaki K (2010). AREB1, AREB2, and ABF3 are master transcription factors that cooperatively regulate ABRE-dependent ABA signaling involved in drought stress tolerance and require ABA for full activation. Plant J.

[33] Zhang C, Li C, Liu J, Lv Y, Yu C, Li H, Zhao T, Liu B (2017). The OsABF1 transcription factor improves drought tolerance by activating the transcription of COR413-TM1 in rice. J Exp Bot.

[34] Fujii H, Verslues PE, Zhu JK (2011). Arabidopsis decuple mutant reveals the importance of SnRK2 kinases in osmotic stress responses in vivo. Proc Natl Acad Sci U S A.

[35] Fujita Y, Nakashima K, Yoshida T, Katagiri T, Kidokoro S, Kanamori N, Umezawa T, Fujita M, Maruyama K, Ishiyama K (2009). Three SnRK2 protein kinases are the main positive regulators of abscisic acid signaling in response to water stress in Arabidopsis. Plant Cell Physiol.

[36] Kobayashi Y, Murata M, Minami H, Yamamoto S, Kagaya Y, Hobo T, Yamamoto A, Hattori T (2005). Abscisic acid-activated SNRK2 protein kinases function in the gene-regulation pathway of ABA signal transduction by phosphorylating ABA response element-binding factors. Plant J.

[37] Kagaya Y, Hobo T, Murata M, Ban A, Hattori T (2002). Abscisic acid-induced transcription is mediated by phosphorylation of an abscisic acid response element binding factor, TRAB1. Plant Cell.

[38] Islam MA, Du H, Ning J, Ye H, Xiong L (2009). Characterization of Glossy1-homologous genes in rice involved in leaf wax accumulation and drought resistance. Plant Mol Biol.

[39] Zhou L, Ni E, Yang J, Zhou H, Liang H, Li J, Jiang D, Wang Z, Liu Z, Zhuang C (2013). Rice OsGL1-6 is involved in leaf cuticular wax accumulation and drought resistance. PLoS One.

[40] Jeong JS, Kim YS, Baek KH, Jung H, Ha SH, Do Choi Y, Kim M, Reuzeau C, Kim JK (2010). Root-specific expression of OsNAC10 improves drought tolerance and grain yield in rice under field drought conditions. Plant Physiol.

[41] Du H, Wang N, Cui F, Li X, Xiao J, Xiong L (2010). Characterization of the beta-carotene hydroxylase gene DSM2 conferring drought and oxidative stress resistance by increasing xanthophylls and abscisic acid synthesis in rice. Plant Physiol.

[42] Zhao T, Liu J, Li HY, Lin JZ, Bian MD, Zhang CY, Zhang YX, Peng YC, Liu B, Lin C (2015). Using hybrid transcription factors to study gene function in rice. Sci China Life Sci.

[43] Ma X, Zhang Q, Zhu Q, Liu W, Chen Y, Qiu R, Wang B, Yang Z, Li H, Lin Y (2015). A robust CRISPR/Cas9 system for convenient, high-efficiency multiplex genome editing in monocot and dicot plants. Mol Plant.

[44] Zhang H, Zhang J, Wei P, Zhang B, Gou F, Feng Z, Mao Y, Yang L, Xu N, Zhu JK (2014). The CRISPR/Cas9 system produces specific and homozygous targeted gene editing in rice in one generation. Plant Biotechnol J.

[45] Xiao B, Huang Y, Tang N, Xiong L (2007). Over-expression of a LEA gene in rice improves drought resistance under the field conditions. Theor Appl Genet.

[46] Yue B, Xue W, Xiong L, Yu X, Luo L, Cui K, Jin D, Xing Y, Zhang Q (2006). Genetic basis of drought resistance at reproductive stage in rice: separation of drought tolerance from drought avoidance. Genetics.

[47] Rao MV, Lee H, Creelman RA, Mullet JE, Davis KR (2000). Jasmonic acid signaling modulates ozone-induced hypersensitive cell death. Plant Cell.

[48] Benedetti CE, Arruda P (2002). Altering the expression of the chlorophyllase gene ATHCOR1 in transgenic Arabidopsis caused changes in the chlorophyll-to-chlorophyllide ratio. Plant Physiol.

[49] Livak KJ, Schmittgen TD (2001). Analysis of relative gene expression data using real-time quantitative PCR and the 2(−Delta Delta C(T)) method. Methods.

[50] Liu L, Zhang Y, Tang S, Zhao Q, Zhang Z, Zhang H, Dong L, Guo H, Xie Q (2010). An efficient system to detect protein ubiquitination by agroinfiltration in Nicotiana benthamiana. Plant J.

