# Supplementary material for: A stress-responsive bZIP transcription factor OsbZIP62 improves drought and oxidative tolerance in rice
Source: BMC Plant Biol. 2019 Jun 17;19:260. doi: 10.1186/s12870-019-1872-1 (PMC6580479; doi:10.1186/s12870-019-1872-1)
Supplement: Supplementary file 1 — Figure S1. Protein sequence alignment of OsbZIP62 and the third subfamily bZIP transcription factors in rice. Figure S2. Molecular phylogenetic tree of the third bZIP subfamily members. Figure S3. Identification of OsbZIP62-VP64 overexpression lines. Figure S4. Characterization of osbzip62 mutants. Figure S5. ABA sensitivity of osbzip62 mutants. (DOCX 2016 kb) [file 12870_2019_1872_MOESM1_ESM.docx]

**Additional files**

**A stress-responsive bZIP transcription factor *OsbZIP62* improves drought and oxidative tolerance in rice**

**Shiqin Yang^1,2†^, Kai Xu^2,†^, Shoujun Chen^2^, Tianfei Li^2^, Hui Xia^2^,Liang Chen^2^, Hongyan Liu^2^，Lijun Luo^1,2*^**


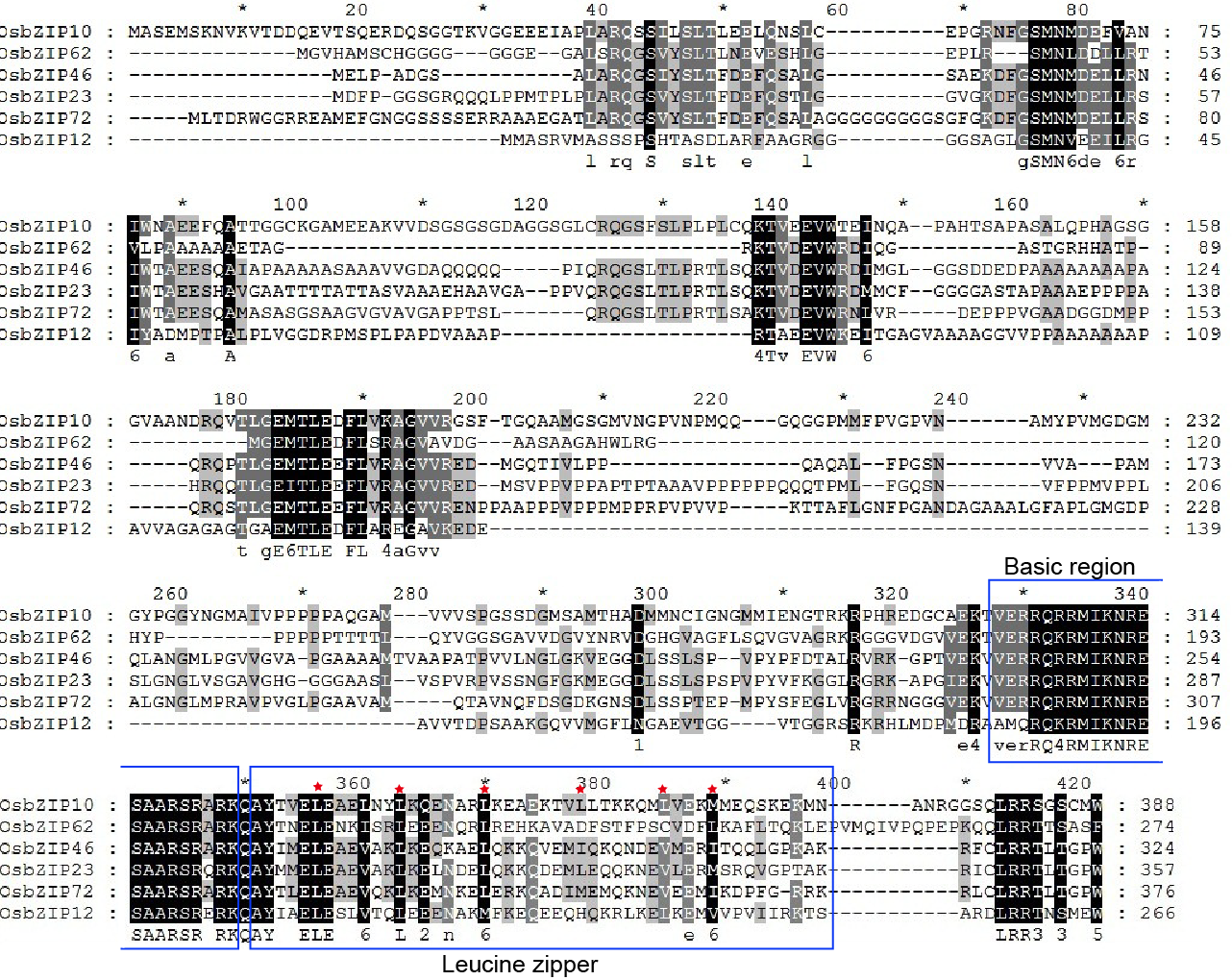


**Figure S1.** Protein sequence alignment of OsbZIP62 and the third subfamily ZIP transcription factors in rice.

Protein sequence alignment of OsbZIP62 with other third subfamily ZIP proteins. The blue boxes indicated the definitive domains, basic region and leucine zipper. The conserved leucine residues were indicated by red stars. The protein accession numbers are as follows: OsbZIP62, LOC_Os07g48660; OsbZIP10, LOC_Os01g64000; OsbZIP12, LOC_Os01g64730; OsbZIP23, LOC_Os02g52780; OsbZIP46, LOC_Os06g10880; OsbZIP72, LOC_Os09g28310

**
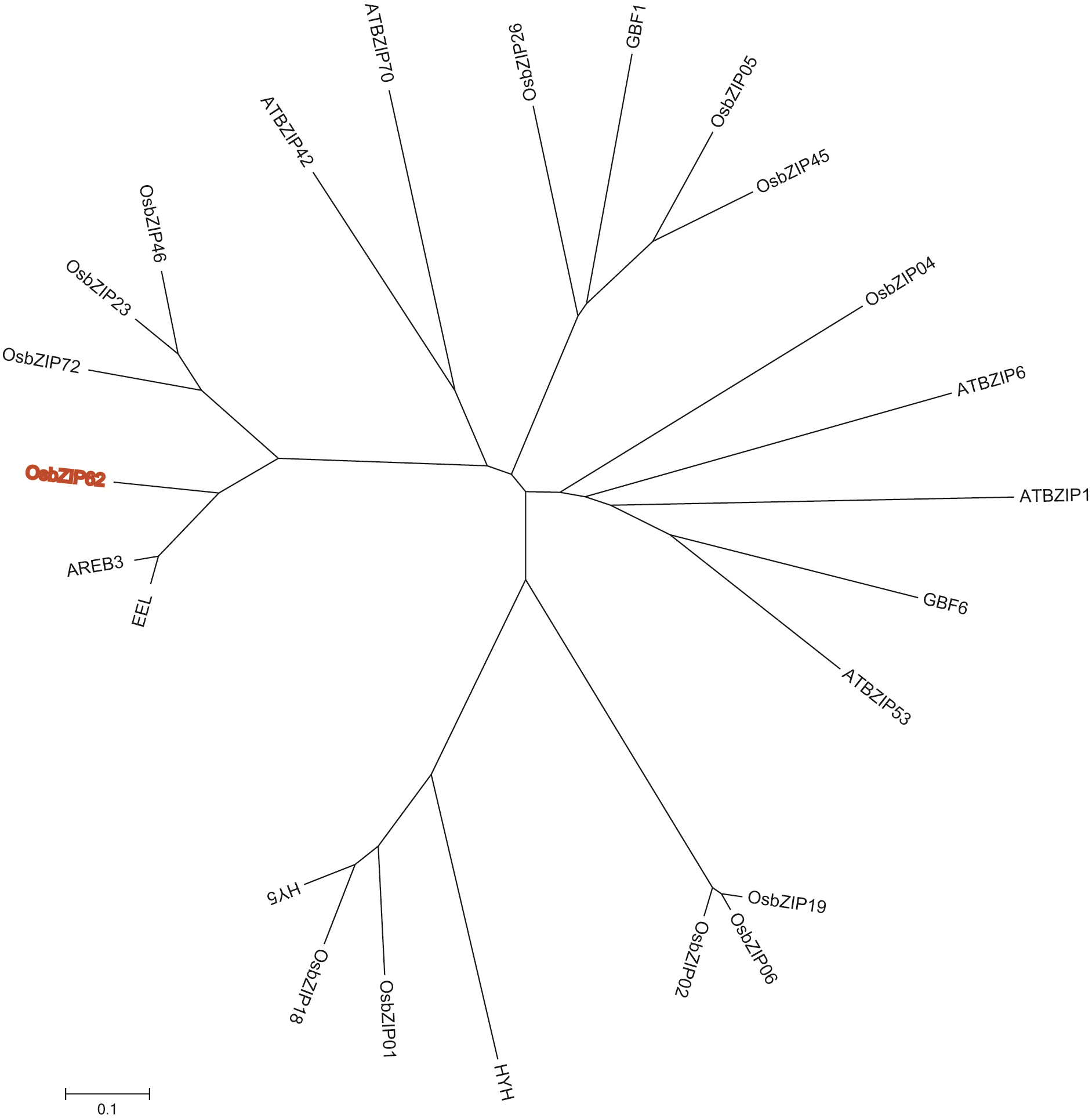
**

**Figure S2.** Molecular phylogenetic tree of the third bZIP subfamily members. Proteins sequences are mainly from Arabidopsis and rice. The bZIP protein sequence alignment and phylogenetic tree construction were performed using the MEGA6 software. The protein accession numbers are as follows: OsbZIP10, LOC_Os01g64000; OsbZIP12, LOC_Os01g64730; EEL, AT2G41070; AREB3, [AT3G56850](http://plntfdb.bio.uni-potsdam.de/v3.0/gene_details.php?pep_id=AT3G56850.1&sp_id=ATH), OsbZIP62, LOC_Os07g48660; OsbZIP72, LOC_Os09g28310; OsbZIP23, LOC_Os02g52780; OsbZIP46, LOC_Os06g10880; ATBZIP42, [AT3G30530](http://plntfdb.bio.uni-potsdam.de/v3.0/gene_details.php?pep_id=AT3G30530.1&sp_id=ATH); ATBZIP70,[AT5G60830](http://plntfdb.bio.uni-potsdam.de/v3.0/gene_details.php?pep_id=AT5G60830.1&sp_id=ATH); OsbZIP26, LOC_Os03g13614; GBF1, [AT4G36730](http://plntfdb.bio.uni-potsdam.de/v3.0/gene_details.php?pep_id=AT4G36730.2&sp_id=ATH); OsbZIP05, LOC_Os01g46970; OsbZIP45, LOC_Os05g49420; OsbZIP04, LOC_Os01g36220; AtbZIP6, AT2G22850; ATBZIP1, [AT5G49450](http://plntfdb.bio.uni-potsdam.de/v3.0/gene_details.php?pep_id=AT5G49450.1&sp_id=ATH); GBF6, AT4G34590; AtbZIP53, AT3G62420; OsbZIP19, LOC_Os02g14910; OsbZIP06, LOC_Os01g55150; OsbZIP02, LOC_Os01g11350; HYH, [AT3G17609](http://plntfdb.bio.uni-potsdam.de/v3.0/gene_details.php?pep_id=AT3G17609.4&sp_id=ATH); OsbZIP01, LOC_Os01g07880; OsbZIP18, LOC_Os02g10860; HY5, AT5G11260


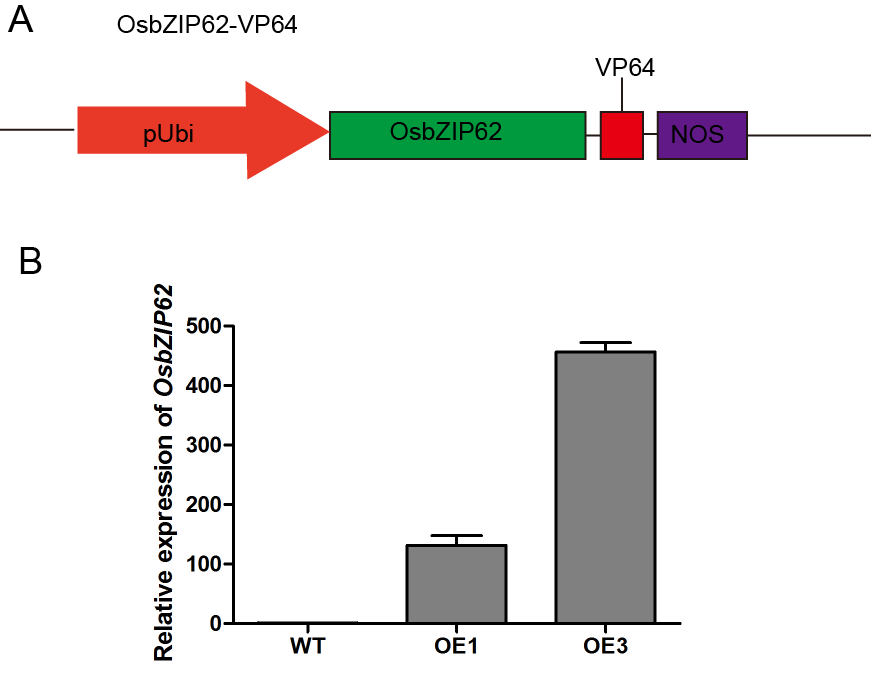


**Figure S3.**  Identification of *OsbZIP62-VP64* overexpression lines.

A, Schematic diagram of *OsbZIP62-VP64* Overexpression construct. B, Expression level analysis of *OsbZIP62* in *OsbZIP62-VP64* Overexpression lines (OE1 and OE3) by qPCR.


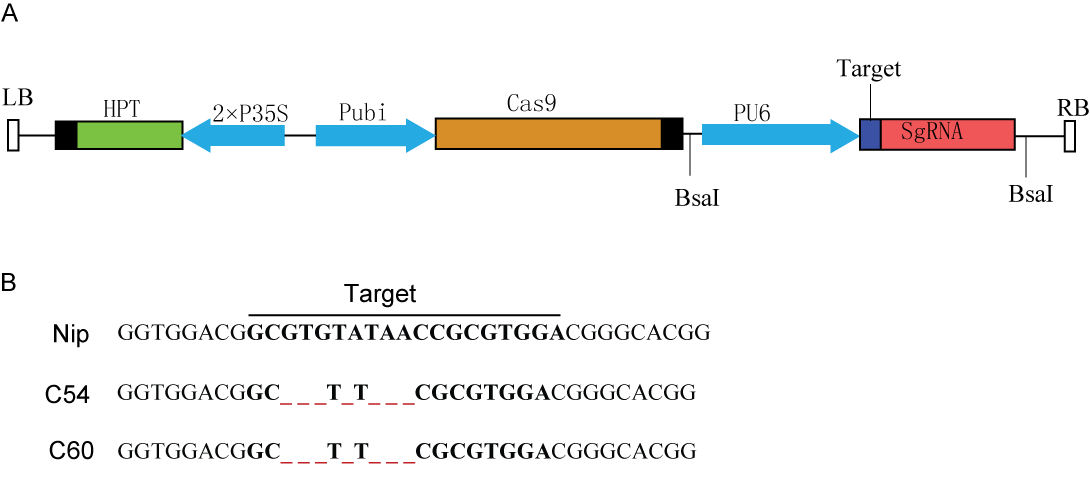


**Figure S4.**  Characterization of *osbzip62* mutants.

A, Schematic diagram of OsbZIP62-CRISPR/Cas9 construct. B, Representative sequence of mutated targets in two homozygous rice plants.


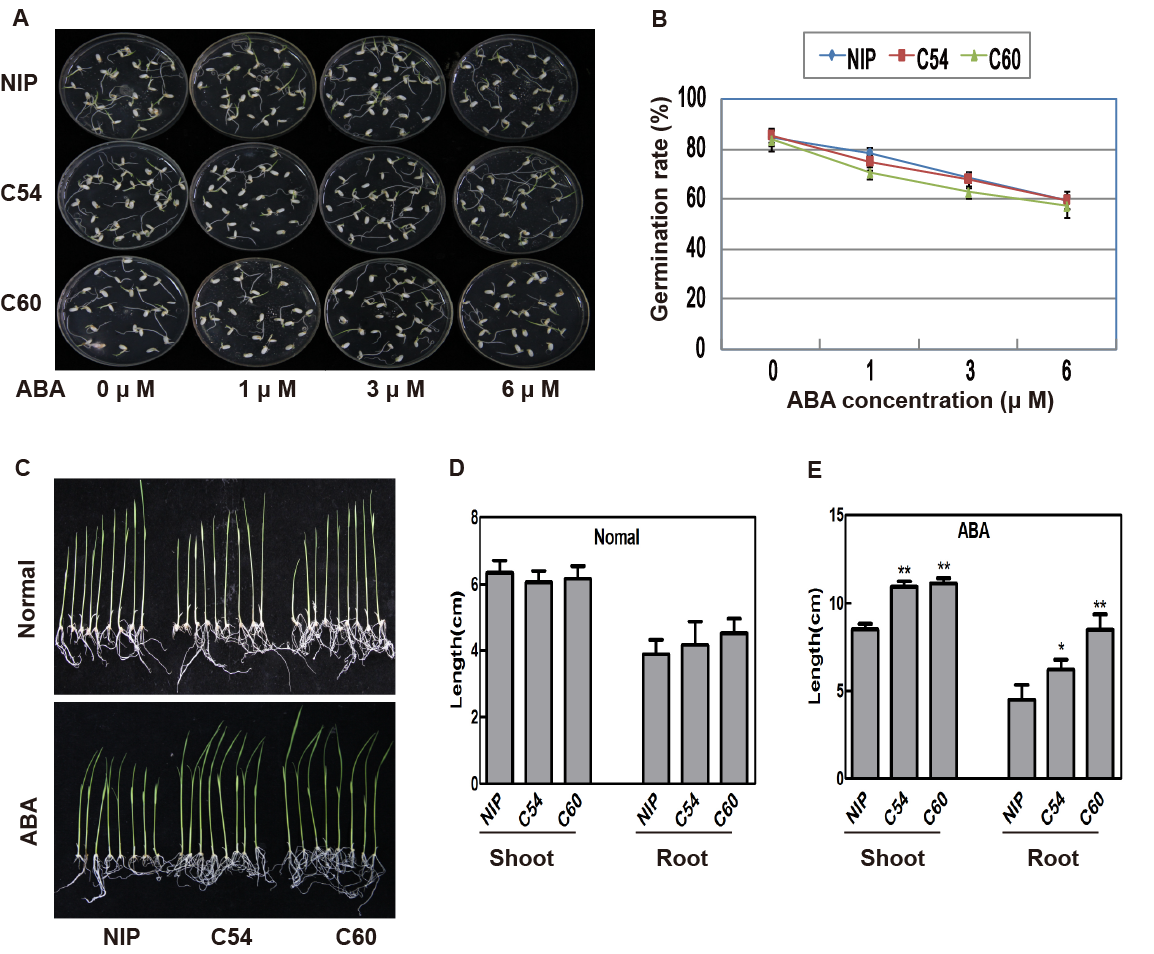


**Figure S5.** ABA sensitivity of *osbzip62* mutant

A, Germination performance of osbzip62-mutant transgenic lines (C54 and C60) and the WT seeds on 1/2MS medium containing 0, 1, 3, 6 μM ABA at 7 d after initiation. B, The germination rate of osbzip62 mutants transgenic seeds (C54 and C60) and the wild-type control plants seeds on 1/2MS medium containing 0, 1, 3, 6 μM ABA. Error bars indicate SE based on three technical replicates.
